# Associations of adiponectin, leptin, and the adiponectin-to-leptin ratio with sarcopenia in older adults with cardiovascular-kidney-metabolic syndrome

**DOI:** 10.1016/j.jnha.2026.100921

**Published:** 2026-07-01

**Authors:** Yan Zhou, Yushan Zhang, Ming Yang, Zehong Huo, Hong Shi, Ji Shen, Chi Zhang

**Affiliations:** a Department of General Practice, Beijing Hospital, National Center for Gerontology, National Clinical Research Center for Gerontology, The Key Laboratory of Geriatrics of NHC, Institute of Geriatric Medicine, Chinese Academy of Medical Sciences, Beijing, 100730, China; b Department of Geriatrics, Beijing Hospital, National Center for Gerontology, National Clinical Research Center for Gerontology, The Key Laboratory of Geriatrics of NHC, Institute of Geriatric Medicine, Chinese Academy of Medical Sciences, Beijing, 100730, China; c Department of Basic Innovation Research, Beijing Hospital, National Center for Gerontology, National Clinical Research Center for Gerontology, The Key Laboratory of Geriatrics of NHC, Beijing Key Laboratory of Aging Mechanism and Intervention Research on Aging-Related Diseases, Institute of Geriatric Medicine, Chinese Academy of Medical Sciences, Beijing, 100730, China

**Keywords:** Cardiovascular-kidney-metabolic syndrome, Adiponectin, Leptin, Sarcopenia

## Abstract

**Objective:**

Adiponectin and leptin are key adipokines associated with adipose tissue and skeletal muscle metabolism. This study aimed to investigate the associations of adiponectin, leptin, and the adiponectin-to-leptin ratio (A/L ratio) with sarcopenia in older adults with cardiovascular-kidney-metabolic (CKM) syndrome.

**Methods:**

This cross-sectional study included 632 older adults (70.60 ± 6.09 years; 56.8% female) with CKM syndrome stages 1–4. Sarcopenia was defined according to the Asian Working Group for Sarcopenia 2019 criteria. Plasma adiponectin and leptin were measured by ELISA and multiplex bead array, and were ln-transformed. Binary and multinomial logistic regression were used to analyze the associations of adiponectin, leptin, and the A/L ratio with sarcopenia, with adjustments for demographic characteristics, BMI, and health status. Receiver operating characteristic curves were used to evaluate the discriminative ability of adipokines.

**Results:**

256 (40.5%) and 57 (9.0%) participants had possible sarcopenia and sarcopenia, respectively. Binary logistic regression revealed that higher adiponectin was independently associated with higher odds of low physical function (OR = 2.11, 95% CI: 1.52–2.98); higher leptin with higher odds of low muscle mass (OR = 1.96, 95% CI: 1.26–3.08) and lower odds of low physical function (OR = 0.65, 95% CI: 0.49–0.87); and a higher A/L ratio with lower odds of low muscle mass (OR = 0.80, 95% CI: 0.65–0.98) but higher odds of low muscle strength (OR = 1.26, 95% CI: 1.06–1.50) and low physical function (OR = 1.24, 95% CI: 1.09–1.42) (all *P* < 0.05). In fully adjusted multinomial logistic regression, adipokines were significantly associated with possible sarcopenia but not with sarcopenia. A/L ratio showed significant AUC values for possible sarcopenia (AUC = 0.641, P < 0.001) and sarcopenia (AUC = 0.617, P = 0.004), with slightly higher performance in CKM stages 1–2 than in stages 3–4.

**Conclusions:**

Adiponectin, leptin, and the A/L ratio exhibit component-specific associations with sarcopenia in older adults with CKM syndrome. These adipokines may help identify sarcopenia status, particularly in early CKM stages.

## 1 Introduction

Cardiovascular-kidney-metabolic (CKM) syndrome, as formally defined by the American Heart Association (AHA) in 2023, represents a systemic pathological continuum arising from the interplay of metabolic risk factors, chronic kidney disease (CKD), and cardiovascular disease (CVD) [1,2]. Epidemiological data indicate that nearly 90% of US adults reside within stages 1 through 4 of CKM syndrome, with over half of adults aged 65 years and older classified at advanced stages [3,4]. Given that adipose tissue dysfunction lies at the pathophysiological core of CKM syndrome, the dysregulation of adipose-derived hormones, particularly adipokines, constitutes a critical molecular bridge linking metabolic dysfunction to downstream organ damage [2,5].

Sarcopenia, defined as the progressive loss of skeletal muscle mass and function in older adults, has emerged as an important comorbidity across the CKM syndrome spectrum. Prior studies have documented that sarcopenia prevalence escalates with advancing CKM stage, with those at stages 3–4 demonstrating more than three-fold increased odds compared with stage 0 [6]. Sarcopenic skeletal muscle amplifies cardiometabolic risk by reducing peripheral glucose uptake, promoting ectopic lipid deposition, and sustaining a pro-inflammatory milieu [[7], [8], [9], [10]]. This bidirectional relationship underscores the necessity of investigating sarcopenia within the CKM framework. Adiponectin and leptin are the two most abundant and well-characterized adipokines, functioning in a reciprocal regulatory axis [5,11]. Under physiological conditions, the adiponectin-to-leptin (A/L) ratio reflects the functional balance of the adipose-endocrine axis and has been proposed as a robust biomarker of adipose tissue dysfunction, cardiometabolic risk, and metabolic syndrome [[12], [13], [14]]. In obese and metabolically dysregulated individuals, this balance shifts, characterized by a decline in adiponectin and an elevation in leptin, thereby predisposing to systemic inflammation, insulin resistance, and organ injury [5].

Despite the established roles of adiponectin and leptin in cardiometabolic homeostasis, their associations with sarcopenia remain incompletely understood, particularly regarding specific sarcopenia components. Meta-analytic evidence indicates that sarcopenia patients exhibit significantly elevated adiponectin levels [15], a finding that appears paradoxical given adiponectin's canonical protective functions. This adiponectin paradox may reflect compensatory hypersecretion in the context of adiponectin resistance, a phenomenon analogous to insulin resistance in metabolic syndrome [16]. Observational studies in older adults have further demonstrated that higher adiponectin levels are associated with lower skeletal muscle mass and greater myosteatosis, even after controlling for adiposity [17,18]. Leptin's relationship with skeletal muscle is equally complex. High leptin levels are associated with reduced muscle mass in the context of leptin resistance and obesity [19,20], its direct effects on muscle strength and physical performance remain less consistent across populations [21,22].

The relationship of adipokines with sarcopenia and its components in individuals affected by CKM syndrome has not been examined. This study aimed to investigate the independent associations of adiponectin, leptin, and the A/L ratio with sarcopenia in older adults with CKM syndrome stages 1–4. We further evaluated the discriminative ability of these adipokines for identifying sarcopenia across different CKM stages.

## 2 Methods

### 2.1 Study sample

This cross-sectional study was conducted between February and August 2023 in five communities in Beijing, China. The inclusion criteria were as follows: (1) age ≥ 60 years; (2) clear consciousness and adequate communication ability; (3) absence of severe psychiatric disorders; and (4) voluntary participation with willingness to complete the questionnaire and functional assessment. A total of 937 older adults were initially enrolled. We excluded 233 participants without available blood samples, 7 participants who did not complete muscle mass, handgrip strength or physical performance assessments, 28 participants with missing adiponectin or leptin data, and 37 participants who did not meet the criteria for CKM syndrome stages 1–4. A total of 632 participants were included in the final analysis. As shown in Supplementary Table S1, we compared core variables between participants without blood specimens and the final sample. Only age, physical activity habit and depressive symptoms differed between these two groups. The study protocol received approval from the ethics committee of Beijing Hospital (No. 2022BJYYEC-260-03). Written informed consent was obtained from all participants before the survey.

### 2.2 Definition of CKM syndrome

In the present study, cardiovascular-kidney-metabolic (CKM) syndrome was defined and stratified into four consecutive stages in full alignment with the 2023 American Heart Association (AHA) statement [2], which established a continuum of disease progression from early pathophysiological alterations to advanced clinical events. Stage 1 represents the initial risk phase of CKM syndrome, defined as the presence of either obesity or impaired fasting glucose (IFG), without concomitant additional metabolic risk factors or chronic kidney disease (CKD). For this study, IFG was operationalized as a fasting plasma glucose level between 5.6 mmol/L and 6.9 mmol/L. Obesity was defined as a body mass index (BMI) of 23 kg/m² or higher, or the presence of abdominal obesity. Abdominal obesity was defined as a waist circumference of 90 cm or greater for male participants and 80 cm or greater for female participants. Stage 2 CKM syndrome was defined as the presence of moderate-to-severe CKD or established metabolic risk factors. Moderate-to-severe CKD was operationalized as an estimated glomerular filtration rate (eGFR) below 60 mL/min/1.73 m². Eligible metabolic risk factors for this stage included hypertension, diabetes mellitus, metabolic syndrome, and hypertriglyceridemia. Hypertriglyceridemia was defined as a fasting serum triglyceride concentration of 1.7 mmol/L or higher. Stage 3 CKM syndrome included individuals with subclinical atherosclerotic cardiovascular disease (ASCVD), high-risk CKD, or elevated 10-year cardiovascular disease (CVD) risk. High-risk CKD was defined as an eGFR below 30 mL/min/1.73 m^2^. The 10-year CVD risk was assessed using the simplified World Health Organization/International Society of Hypertension (WHO/ISH) risk prediction score, and a score of 5 or higher was classified as high-risk status. Stage 4, the most advanced phase of CKM syndrome, was defined as clinically confirmed CVD accompanied by excess adiposity, metabolic risk factors, or CKD. Clinically confirmed CVD conditions eligible for this stage included coronary heart disease, heart failure, stroke, and peripheral artery disease.

### 2.3 Blood sample collection and laboratory examination

All eligible participants provided 5 mL fasting venous blood samples after an overnight fast, with collection completed prior to morning meal intake. The collected blood samples were immediately treated with anticoagulant, and then subjected to centrifugation at 2000 × g for 10 min under a constant 4℃ condition. Plasma specimens were isolated from the centrifuged samples, aliquoted, and preserved at −80℃ in an ultra-low temperature freezer until subsequent laboratory analysis. Routine biochemical parameters required for the diagnosis and staging of CKM syndrome were measured using a fully automated biochemical analyzer. The tested indicators included total cholesterol (TC), triglycerides (TG), high-density lipoprotein cholesterol (HDL-C), low-density lipoprotein cholesterol (LDL-C), fasting plasma glucose (FPG), and creatinine. The estimated glomerular filtration rate (eGFR) for each participant was calculated using the Chronic Kidney Disease Epidemiology Collaboration (CKD-EPI) formula. Plasma adiponectin and leptin concentrations were detected using commercial detection kits (RayBiotech, US). Specifically, plasma adiponectin level was measured via enzyme-linked immunosorbent assay (ELISA), and plasma leptin level was quantified using a customized human multiplex bead-based immunoassay. For both detection assays, the intra-assay and inter-assay coefficients of variation (CVs) were all below 10%. Both adiponectin and leptin exhibited unimodal skewed distributions. Accordingly, these two adipokines as well as the A/L ratio were subjected to natural logarithmic transformation prior to multivariate statistical analyses.

### 2.4 Assessment of sarcopenia

Sarcopenia was assessed according to the 2019 Asian Working Group for Sarcopenia (AWGS 2019) consensus criteria [23]. Muscle mass was evaluated by appendicular skeletal muscle mass measured with bioelectrical impedance analysis (Inbody 770, South Korea). Appendicular skeletal muscle mass index (SMI) was calculated as appendicular skeletal muscle mass divided by height squared. Low muscle mass was defined as SMI below 7.0 kg/m² for men and below 5.7 kg/m² for women. Muscle strength was evaluated by handgrip strength measured with a calibrated handheld dynamometer. Low muscle strength was defined as below 28 kg for men and below 18 kg for women. Physical performance was evaluated using the 6-meter gait speed test, 5-time sit-to-stand test (5TSTS), and the Short Physical Performance Battery (SPPB), in accordance with the 2019 Asian Working Group for Sarcopenia (AWGS 2019) consensus. Low physical performance was defined as meeting any one of the following criteria: gait speed slower than 1.0 m/s, 5TSTS of 12 seconds or longer, or SPPB score ≤ 9 points. Participants were classified into three groups: non-sarcopenia, possible sarcopenia (low muscle strength and/or low physical performance with normal muscle mass), and sarcopenia (low muscle mass plus low muscle strength and/or low physical performance).

### 2.5 Covariate

A set of covariates were selected for multivariable analyses based on prior relevant literature and clinical relevance. Trained investigators conducted face-to-face interviews to collect demographic characteristics and lifestyle information, including age, sex, ethnicity, marital status, current smoking, and current alcohol consumption. Height and weight were measured using standardized procedures to calculate BMI. Activities of daily living (ADL) were evaluated with the Barthel Index, and ADL impairment was defined as a total score < 95. Cognitive impairment was assessed using the Mini-cog test, which consists of a 3-word recall task and the clock drawing test. Depressive symptoms were measured with the Patient Health Questionnaire-9 (PHQ-9), and a total score > 4 indicated the presence of depressive symptoms. Polypharmacy was defined according to the number of regularly used prescription medications. In addition, biochemical parameters related to CKM syndrome were included as covariates.

### 2.6 Statistical analysis

Continuous variables were expressed as mean ± standard deviation (SD) for normally distributed data or median (interquartile range) for non-normally distributed data. Categorical variables were expressed as frequencies and percentages. Differences among the three sarcopenia groups were examined using one-way analysis of variance (ANOVA) or Kruskal-Wallis H test for continuous variables and chi-square test for categorical variables. Spearman correlation coefficients were calculated to examine the relationship of adiponectin, leptin, and the A/L ratio with sarcopenia indicators. Linear regression models were employed to evaluate the associations of adiponectin, leptin, and the A/L ratio with sarcopenia indicators (SMI, handgrip strength, 5TSTS, 6-meter gait speed, and SPPB score). Binary Logistic regression was used to assess the associations for the presence of sarcopenia components. Multinomial logistic regression was performed to analyze the relationships between adipocytokines and sarcopenia severity with non-sarcopenia group as the reference. Demographic characteristics, BMI, health status, and biochemical indicators were sequentially included into the multivariable models, and odds ratios (OR) with 95% confidence intervals (CI) were calculated. Receiver operating characteristic (ROC) curve analyses were conducted to evaluate the discriminatory ability of adiponectin, leptin, and the A/L ratio for identifying sarcopenia. The area under the curve (AUC) with 95% confidence interval (CI) was calculated, and the optimal cut-off value was determined by the Youden index. Subgroup ROC analyses were conducted stratified by CKM stage (stages 1–2 vs. stages 3–4).

Several sensitivity analyses were performed to verify the robustness of the main findings. First, to address the potential heterogeneity in the reference group caused by isolated low muscle mass in the non-sarcopenia group, all regression models were rerun after excluding participants with isolated low muscle mass who had normal muscle strength and physical performance. Second, to evaluate the rationale of BMI adjustment and assess the total associations between adipokines and sarcopenia related outcomes, fully adjusted models without BMI as a covariate were constructed. In addition, stratified regression analyses by CKM stage were conducted, and interaction terms between adipokines and CKM stage were added to the overall models to test for effect modification. CKM stages were grouped into early (stages 1−2) and advanced (stages 3−4) based on disease severity and sample size considerations, as individual stages 1 and 4 had relatively small sample sizes. All analyses were performed using R software (version 4.3.1; R Foundation for Statistical Computing, Vienna, Austria). A two-sided P < 0.05 was considered statistically significant.

## 3 Results

### 3.1 Participant characteristics

**Fig. 1:**
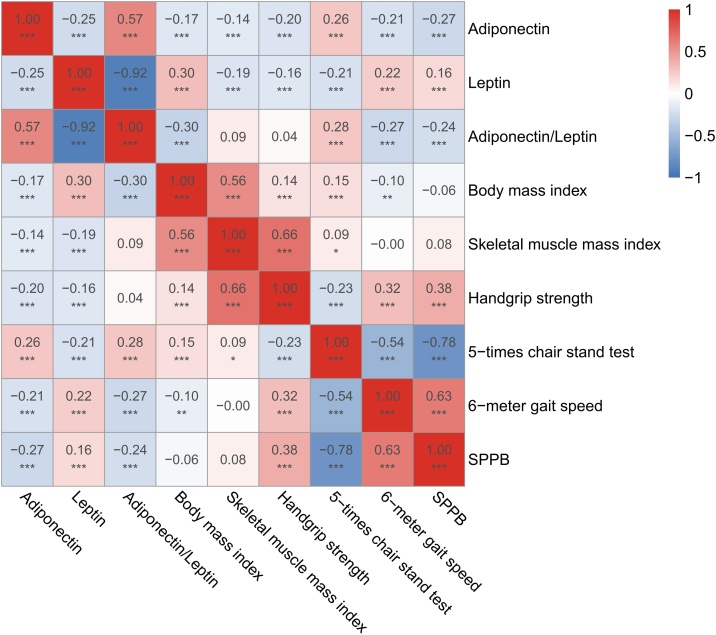
Correlation heatmap of adiponectin, leptin, adiponectin-to-leptin ratio with sarcopenia indicators.

Among the 632 included older adults with CKM syndrome, the mean age was 70.60 ± 6.09 years, and 56.8% were female. The prevalence of possible sarcopenia and sarcopenia were 40.5% (n = 256) and 9.0% (n = 57), respectively. According to the 2019 AWGS criteria, 48 participants in the non-sarcopenia group presented with isolated low muscle mass without concomitant low muscle strength or impaired physical performance. The prevalence of ADL impairment, cognitive impairment, and depressive symptoms increased significantly across sarcopenia severity (all P < 0.001). Adiponectin levels were significantly higher in the possible sarcopenia and sarcopenia groups compared with the non-sarcopenia group (P < 0.001). Conversely, leptin levels were significantly lower in the sarcopenia group (P < 0.001). The A/L ratio was significantly higher in the possible sarcopenia and sarcopenia groups (P < 0.001). The detailed baseline characteristics are presented in Table 1. As shown in Fig. 1, Adiponectin was negatively correlated with SMI (r = −0.14), handgrip strength (r = −0.20), 6-meter gait speed (r = −0.21), and SPPB score (r = −0.27), and positively correlated with 5TSTS (r = 0.26); while leptin was negatively correlated with SMI (r = −0.19), handgrip strength (r = −0.16), and 5TSTS (r = −0.21), and positively correlated with 6-meter gait speed (r = 0.22) and SPPB score (r = 0.16) with all P < 0.001. A/L ratio was positively correlated with 5TSTS (r = 0.28), and was negatively correlated with 6-meter gait speed (r = −0.27) and SPPB score (r = −0.24) (all P < 0.001). Additionally, BMI was negatively correlated with adiponectin (r = −0.17, P < 0.001) and positively correlated with leptin (r = 0.30, P < 0.001).

**Table 1:** Sample characteristics of the 632 included older adults with CKM syndrome by sarcopenia status.

Variable	Total (N = 632)	Non-sarcopenia (n = 319)	Possible sarcopenia (n = 256)	Sarcopenia (n = 57)	P-value
Sample characteristics					
Age, years, mean ± SD	70.60 ± 6.09	68.36 ± 5.35	72.36 ± 5.70	75.26 ± 6.56	<0.001
Female, n (%)	359 (56.8)	181 (56.7)	142 (55.5)	36 (63.2)	0.570
Han ethnicity, n (%)	592 (93.7)	300 (94.0)	238 (93.0)	54 (94.7)	0.820
Education level, n (%)					<0.001
Primary or below	23 (3.6)	4 (1.3)	14 (5.5)	5 (8.8)	
Secondary	422 (66.8)	198 (62.1)	186 (72.7)	38 (66.7)	
Tertiary or above	187 (29.6)	117 (36.7)	56 (21.9)	14 (24.6)	
Married and cohabiting, n (%)	535 (84.7)	289 (90.6)	200 (78.1)	46 (80.7)	<0.001
BMI, kg/m², mean ± SD	24.89 ± 3.35	24.64 ± 3.25	25.81 ± 3.16	22.14 ± 2.98	<0.001
Current smoking, n (%)	73 (11.6)	37 (11.6)	31 (12.1)	5 (8.8)	0.775
Current drinking, n (%)	73 (11.6)	33 (10.3)	33 (12.9)	7 (12.3)	0.627
Physical activity habit, n (%)	540 (85.4)	281 (88.1)	214 (83.6)	45 (78.9)	0.109
Health status					
ADL impairment, n (%)	39 (6.2)	9 (2.8)	22 (8.6)	8 (14.0)	<0.001
Cognitive impairment, n (%)	158 (25.0)	53 (16.6)	79 (30.9)	26 (45.6)	<0.001
Depression, n (%)	87 (13.8)	29 (9.1)	43 (16.8)	15 (26.3)	<0.001
Medication use, M (P_25_, P_75_)	3 (2, 5)	2 (1, 4)	3 (2, 5)	3 (2, 6)	<0.001
Sarcopenia components					
SMI, kg/m², mean ± SD	6.95 ± 1.06	6.96 ± 1.09	7.20 ± 0.90	5.77 ± 0.74	<0.001
Handgrip strength, kg, mean ± SD	28.25 ± 9.24	31.18 ± 9.08	26.09 ± 8.39	21.55 ± 7.63	<0.001
5TSTS, s, mean ± SD	10.99 ± 3.48	8.72 ± 1.69	13.48 ± 3.36	12.50 ± 3.04	<0.001
6-m gait speed, m/s, mean ± SD	1.03 ± 0.23	1.14 ± 0.19	0.91 ± 0.20	0.94 ± 0.21	<0.001
SPPB score, M (P_25_, P_75_)	10 (8, 10)	10 (10, 11)	8 (8, 9)	8 (7, 9)	<0.001
Biomarkers					
TC, mmol/L, mean ± SD	5.32 ± 1.34	5.56 ± 1.37	4.95 ± 1.18	5.61 ± 1.45	<0.001
TG, mmol/L, M (P_25_, P_75_)	1.59 (1.16, 2.55)	1.79 (1.21, 3.02)	1.45 (1.11, 2.17)	1.51 (1.21, 2.08)	<0.001
HDL-C, mmol/L, mean ± SD	1.43 ± 0.43	1.43 ± 0.48	1.42 ± 0.34	1.53 ± 0.42	0.199
LDL-C, mmol/L, mean ± SD	2.89 ± 0.96	2.96 ± 0.98	2.75 ± 0.87	3.17 ± 1.13	0.003
FPG, mmol/L, mean ± SD	4.68 ± 2.67	4.24 ± 2.63	5.28 ± 2.58	4.46 ± 2.77	<0.001
Creatinine, μmol/L, mean ± SD	80.52 ± 40.18	83.26 ± 46.93	76.56 ± 27.42	83.02 ± 46.24	0.123
eGFR, mL/min/1.73m², mean ± SD	77.36 ± 18.75	77.86 ± 19.95	77.69 ± 16.76	73.16 ± 20.09	0.206
Adiponectin, mg/L, M (P_25_, P_75_)	8.76 (5.48, 12.81)	7.03 (4.56, 10.84)	10.00 (6.68, 14.49)	11.26 (7.17, 18.69)	<0.001
Leptin, ng/mL, M (P_25_, P_75_)	1.88 (0.54, 4.51)	2.47 (0.78, 5.04)	1.24 (0.47, 3.45)	0.91 (0.25, 4.10)	<0.001
A/L ratio, M (P_25_, P_75_)	4.48 (1.44, 20.20)	2.65 (1.00, 11.98)	7.10 (2.75, 22.65)	6.97 (2.86, 64.93)	<0.001
CKM stages					<0.001
1	43 (6.8)	33 (10.3)	8 (3.1)	2 (3.5)	
2	357 (56.5)	200 (62.7)	135 (52.7)	22 (38.6)	
3	128 (20.2)	53 (16.6)	54 (21.1)	21 (36.8)	
4	104 (16.5)	33 (10.4)	59 (20.1)	12 (21.1)	

Notes: P-values were obtained using one-way analysis of variance, Kruskal-Wallis rank sum test, or chi-square test, as appropriate.

ADL, activities of daily living; A/L ratio, adiponectin-to-leptin ratio; BMI, body mass index; CKM, cardiovascular-kidney-metabolic; eGFR, estimated glomerular filtration rate; FPG, fasting plasma glucose; HDL-C, high-density lipoprotein cholesterol; LDL-C, low-density lipoprotein cholesterol; SMI, skeletal muscle mass index; SPPB, Short Physical Performance Battery; TC, total cholesterol; TG, triglycerides; 5TSTS, 5-time sit-to-stand test.

### 3.2 Linear regression analyses of adipokines, leptin, and A/L ratio with SMI, muscle strength, and physical function

Table 2 presents the linear regression results for the associations of adiponectin, leptin, and the A/L ratio with sarcopenia components across all adjusted models in the total sample. In the fully adjusted model (Model 4), higher adiponectin levels were significantly associated with longer 5TSTS (β = 0.704, P < 0.001), slower gait speed (β = −0.026, P = 0.046), and lower SPPB score (β = −0.277, P = 0.002), but not with SMI or handgrip strength. Higher leptin levels were significantly associated with lower SMI (β = −0.237, P < 0.001), faster gait speed (β = 0.063, P < 0.001), and higher SPPB score (β = 0.178, P = 0.034). The A/L ratio demonstrated the most consistent associations across all sarcopenia components in Model 4, with SMI (β = 0.084, P < 0.001), handgrip strength (β = −0.019, P = 0.006), 5TSTS (β = 0.239, P = 0.007), gait speed (β = −0.023, P < 0.001), and SPPB score (β = −0.248, P = 0.003).

**Table 2:** Associations of adiponectin, leptin, and A/L ratio with sarcopenia indicators.

Variable	SMI		Grip strength		5TSTS		Gait speed		SPPB	
	β (95% CI)	P-value	β (95% CI)	P-value	β (95% CI)	P-value	β (95% CI)	P-value	β (95% CI)	P-value
**Ln-Adiponectin**										
Model 1	−0.083 (−0.171, 0.004)	0.063	−0.007 (−0.036, 0.023)	0.645	0.783 (0.391, 1.175)	<0.001	−0.030 (−0.055, −0.005)	0.018	−0.318 (−0.490, −0.146)	<0.001
Model 2	0.028 (−0.035, 0.091)	0.384	−0.013 (−0.041, 0.016)	0.396	0.791 (0.411, 1.171)	<0.001	−0.028 (−0.052, −0.004)	0.024	−0.280 (−0.449, −0.111)	0.001
Model 3	0.028 (−0.034, 0.090)	0.377	−0.012 (−0.041, 0.017)	0.406	0.775 (0.402, 1.148)	<0.001	−0.028 (−0.052, −0.004)	0.020	−0.270 (−0.434, −0.106)	0.001
Model 4	0.032 (−0.036, 0.100)	0.354	−0.022 (−0.054, 0.009)	0.159	0.704 (0.301, 1.107)	<0.001	−0.026 (−0.052, −0.001)	0.046	−0.277 (−0.455, −0.100)	0.002
**Ln-Leptin**										
Model 1	0.056 (−0.021, 0.134)	0.155	0.013 (−0.013, 0.040)	0.310	−0.448 (−0.795, −0.100)	0.012	0.055 (0.034, 0.077)	<0.001	0.230 (0.077, 0.382)	0.003
Model 2	−0.255 (−0.312, −0.198)	<0.001	0.046 (0.018, 0.073)	0.001	−0.769 (−1.132, −0.406)	<0.001	0.073 (0.050, 0.095)	<0.001	0.291 (0.129, 0.453)	<0.001
Model 3	−0.251 (−0.307, −0.194)	<0.001	0.047 (0.019, 0.074)	<0.001	−0.808 (−1.165, −0.450)	<0.001	0.075 (0.053, 0.097)	<0.001	0.303 (0.146, 0.460)	<0.001
Model 4	−0.237 (−0.297, −0.177)	<0.001	0.027 (−0.002, 0.056)	0.063	−0.494 (−0.868, −0.121)	0.010	0.063 (0.040, 0.087)	<0.001	0.178 (0.013, 0.342)	0.034
**Ln-A/L ratio**										
Model 1	−0.039 (−0.075, −0.003)	0.035	−0.010 (−0.022, 0.003)	0.120	0.243 (0.082, 0.405)	0.003	−0.022 (−0.032, −0.012)	<0.001	−0.308 (−0.451, −0.165)	<0.001
Model 2	0.092 (0.065, 0.119)	<0.001	−0.024 (−0.036, −0.011)	<0.001	0.379 (0.212, 0.545)	<0.001	−0.029 (−0.039, −0.018)	<0.001	−0.273 (−0.415, −0.131)	<0.001
Model 3	0.090 (0.063, 0.116)	<0.001	−0.025 (−0.037, −0.012)	<0.001	0.399 (0.235, 0.562)	<0.001	−0.030 (−0.041, −0.020)	<0.001	−0.267 (−0.408, −0.126)	<0.001
Model 4	0.084 (0.056, 0.113)	<0.001	−0.019 (−0.032, −0.006)	0.006	0.239 (0.066, 0.413)	0.007	−0.023 (−0.034, −0.012)	<0.001	−0.248 (−0.407, −0.089)	0.003

Model 1: adjusted for age and sex. Model 2: additionally adjusted for ethnicity, education level, marital status, smoking, alcohol consumption, physical activity, and BMI. Model 3: additionally adjusted for ADL impairment, cognitive impairment, depressive symptoms, and polypharmacy. Model 4: additionally adjusted for TC, TG, HDL-C, LDL-C, FPG, and eGFR.

A/L ratio, adiponectin-to-leptin ratio; CI, confidence interval; SMI, skeletal muscle mass index; SPPB, Short Physical Performance Battery; 5TSTS, 5-time sit-to-stand test.

### 3.3 Binary logistic regression analysis of adipokines, leptin, and A/L ratio with sarcopenia components

Table 3 presents the binary logistic regression results for the associations of adiponectin, leptin, and the A/L ratio with low muscle mass, low muscle strength, and low physical function in the total sample. In the fully adjusted model (Model 4), higher adiponectin levels were significantly associated with higher odds of low physical function (OR: 2.11, 95% CI: 1.52–2.98, P < 0.001), but not with low muscle mass (OR: 0.83, 95% CI: 0.53–1.29, P = 0.395) or low muscle strength (OR: 1.51, 95% CI: 0.99–2.33, P = 0.060). Higher leptin levels were significantly associated with higher odds of low muscle mass (OR: 1.96, 95% CI: 1.26–3.08, P = 0.003) and lower odds of low physical function (OR: 0.65, 95% CI: 0.49–0.87, P = 0.004), but not with low muscle strength (OR: 0.71, 95% CI: 0.48–1.04, P = 0.078). The A/L ratio showed significant associations with all three components in Model 4: lower odds of low muscle mass (OR: 0.80, 95% CI: 0.65–0.98, P = 0.032), and higher odds of low muscle strength (OR: 1.26, 95% CI: 1.06–1.50, P = 0.007) and low physical function (OR: 1.24, 95% CI: 1.09–1.42, P = 0.002). Across multiple models, all three independent variables showed consistent significant associations with low physical function. Regarding low muscle mass, neither leptin nor the A/L ratio showed significant associations in Model 1. However, after additional adjustment for BMI and lifestyle factors (Model 2), both became significantly associated, with leptin showing higher odds (P < 0.001) and the A/L ratio showing lower odds (P < 0.001), and these associations remained significant in Models 3 and 4. In contrast, adiponectin was not significantly associated with low muscle mass in any model (all P > 0.05).

**Table 3:** Binary logistic regression analysis of adipokines and sarcopenia components.

Independent variable	Low muscle mass			Low muscle strength			Low physical function		
	β (95% CI)	OR (95% CI)	P-value	β (95% CI)	OR (95% CI)	P-value	β (95% CI)	OR (95% CI)	P-value
**Ln-Adiponectin**									
Model 1	0.137 (−0.189, 0.472)	1.15 (0.83, 1.60)	0.417	0.325 (−0.020, 0.682)	1.38 (0.98, 1.98)	0.070	0.642 (0.369, 0.926)	1.90 (1.45, 2.52)	<0.001
Model 2	−0.281 (−0.654, 0.092)	0.76 (0.52, 1.10)	0.139	0.255 (−0.106, 0.628)	1.29 (0.90, 1.87)	0.173	0.664 (0.379, 0.961)	1.94 (1.46, 2.61)	<0.001
Model 3	−0.272 (−0.649, 0.105)	0.76 (0.52, 1.11)	0.156	0.257 (−0.106, 0.634)	1.29 (0.90, 1.88)	0.172	0.683 (0.394, 0.984)	1.98 (1.48, 2.67)	<0.001
Model 4	−0.191 (−0.630, 0.252)	0.83 (0.53, 1.29)	0.395	0.410 (−0.010, 0.846)	1.51 (0.99, 2.33)	0.060	0.748 (0.416, 1.091)	2.11 (1.52, 2.98)	<0.001
**Ln-Leptin**									
Model 1	−0.084 (−0.371, 0.199)	0.92 (0.69, 1.22)	0.564	−0.302 (−0.618, 0.003)	0.74 (0.54, 1.00)	0.056	−0.425 (−0.659, −0.194)	0.65 (0.52, 0.82)	<0.001
Model 2	0.922 (0.528, 1.331)	2.51 (1.70, 3.78)	<0.001	−0.267 (−0.620, 0.077)	0.77 (0.54, 1.08)	0.133	−0.593 (−0.864, −0.329)	0.55 (0.42, 0.72)	<0.001
Model 3	0.904 (0.506, 1.318)	2.47 (1.66, 3.74)	<0.001	−0.323 (−0.688, 0.032)	0.72 (0.50, 1.03)	0.078	−0.616 (−0.893, −0.347)	0.54 (0.41, 0.71)	<0.001
Model 4	0.673 (0.230, 1.126)	1.96 (1.26, 3.08)	0.003	−0.344 (−0.733, 0.034)	0.71 (0.48, 1.04)	0.078	−0.427 (−0.722, −0.135)	0.65 (0.49, 0.87)	0.004
**Ln-A/L ratio**									
Model 1	0.066 (−0.070, 0.197)	1.07 (0.93, 1.22)	0.329	0.164 (0.029, 0.296)	1.18 (1.03, 1.34)	0.016	0.224 (0.116, 0.335)	1.25 (1.12, 1.40)	<0.001
Model 2	−0.355 (−0.549, −0.175)	0.70 (0.58, 0.84)	<0.001	0.158 (0.004, 0.309)	1.17 (1.00, 1.36)	0.041	0.292 (0.172, 0.417)	1.34 (1.19, 1.52)	<0.001
Model 3	−0.340 (−0.536, −0.158)	0.71 (0.59, 0.85)	<0.001	0.194 (0.036, 0.350)	1.21 (1.04, 1.42)	0.015	0.308 (0.186, 0.434)	1.36 (1.20, 1.54)	<0.001
Model 4	−0.223 (−0.433, −0.024)	0.80 (0.65, 0.98)	0.032	0.234 (0.062, 0.405)	1.26 (1.06, 1.50)	0.007	0.216 (0.084, 0.352)	1.24 (1.09, 1.42)	0.002

Model 1: adjusted for age and sex. Model 2: additionally adjusted for ethnicity, education level, marital status, smoking, alcohol consumption, physical activity, and BMI. Model 3: additionally adjusted for ADL impairment, cognitive impairment, depressive symptoms, and polypharmacy. Model 4: additionally adjusted for TC, TG, HDL-C, LDL-C, FPG, and eGFR.

A/L ratio, adiponectin-to-leptin ratio; OR, odds ratio; CI, confidence interval.

### 3.4 Multinomial logistic regression analysis of adipokines, leptin, and A/L ratio with sarcopenia severity

Multinomial logistic regression analysis of adipokines, leptin, and A/L ratio with sarcopenia severity was presented in Table 4. Compared with non-sarcopenia, adiponectin, leptin, and the A/L ratio were all significantly associated with possible sarcopenia across all models. In the fully adjusted Model 4, higher adiponectin (OR: 2.08, 95% CI: 1.46–2.96) and a higher A/L ratio (OR: 1.25, 95% CI: 1.09–1.45) were associated with increased odds of possible sarcopenia, while higher leptin was associated with decreased odds (OR: 0.63, 95% CI: 0.46–0.85). In Model 1 (adjusting only for age and sex), adiponectin (OR: 2.05, 95% CI: 1.27–3.33, P = 0.004) and the A/L ratio (OR: 1.42, 95% CI: 1.19–1.70, P < 0.001) were associated with higher odds of sarcopenia, whereas leptin was associated with lower odds (OR: 0.55, 95% CI: 0.36–0.85, P = 0.006). However, after full adjustment for demographic characteristics, lifestyle factors, BMI, health status, and biochemical parameters (Model 4), none of the three biomarkers remained significantly associated with sarcopenia (all P > 0.05).

**Table 4:** Multinomial logistic regression analysis of adipokines and sarcopenia severity.

Independent variable	Possible sarcopenia vs. non-sarcopenia			Sarcopenia vs. non-sarcopenia		
	β (95% CI)	OR (95% CI)	P-value	β (95% CI)	OR (95% CI)	P-value
**Ln-Adiponectin**						
Model 1	0.565 (0.282, 0.849)	1.76 (1.33, 2.34)	<0.001	0.719 (0.235, 1.203)	2.05 (1.27, 3.33)	0.004
Model 2	0.629 (0.327, 0.932)	1.88 (1.39, 2.54)	<0.001	0.362 (−0.122, 0.846)	1.44 (0.88, 2.33)	0.143
Model 3	0.649 (0.343, 0.955)	1.91 (1.41, 2.60)	<0.001	0.408 (−0.084, 0.900)	1.50 (0.92, 2.46)	0.104
Model 4	0.733 (0.382, 1.085)	2.08 (1.46, 2.96)	<0.001	0.534 (−0.043, 1.112)	1.71 (0.96, 3.04)	0.070
**Ln-Leptin**						
Model 1	−0.381 (−0.622, −0.141)	0.68 (0.54, 0.87)	0.002	−0.594 (−1.020, −0.168)	0.55 (0.36, 0.85)	0.006
Model 2	−0.654 (−0.938, −0.371)	0.52 (0.39, 0.69)	<0.001	−0.082 (−0.563, 0.398)	0.92 (0.57, 1.49)	0.737
Model 3	−0.663 (−0.950, −0.376)	0.52 (0.39, 0.69)	<0.001	−0.170 (−0.668, 0.328)	0.84 (0.51, 1.39)	0.503
Model 4	−0.467 (−0.774, −0.159)	0.63 (0.46, 0.85)	0.003	−0.252 (−0.804, 0.299)	0.78 (0.45, 1.35)	0.370
**Ln-A/L ratio**						
Model 1	0.206 (0.091, 0.321)	1.23 (1.10, 1.38)	<0.001	0.353 (0.175, 0.530)	1.42 (1.19, 1.70)	<0.001
Model 2	0.312 (0.182, 0.442)	1.37 (1.20, 1.56)	<0.001	0.141 (−0.063, 0.346)	1.15 (0.94, 1.41)	0.175
Model 3	0.321 (0.190, 0.452)	1.38 (1.21, 1.57)	<0.001	0.189 (−0.023, 0.400)	1.21 (0.98, 1.49)	0.080
Model 4	0.227 (0.085, 0.369)	1.25 (1.09, 1.45)	0.002	0.218 (−0.016, 0.452)	1.24 (0.98, 1.57)	0.068

Model 1: adjusted for age and sex. Model 2: additionally adjusted for ethnicity, education level, marital status, smoking, alcohol consumption, physical activity, and BMI. Model 3: additionally adjusted for ADL impairment, cognitive impairment, depressive symptoms, and polypharmacy. Model 4: additionally adjusted for TC, TG, HDL-C, LDL-C, FPG, and eGFR.

A/L ratio, adiponectin-to-leptin ratio; OR, odds ratio; CI, confidence interval.

### 3.5 ROC curve analysis

**Fig. 2:**
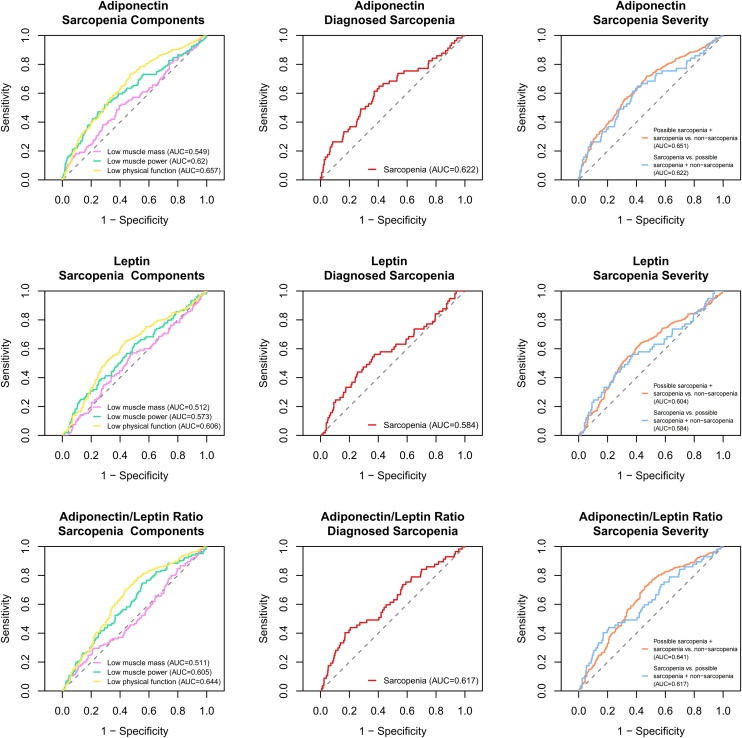
ROC curves of adiponectin, leptin, and the A/L ratio for identifying sarcopenia.

The results of the ROC curve analysis are summarized in Table 5 and Fig. 2. For distinguishing possible sarcopenia in the total sample, the AUC of adiponectin, leptin, and A/L ratio were 0.651 (95% CI: 0.609–0.694), 0.604 (95% CI: 0.560–0.648), and 0.641 (95% CI: 0.598–0.685), respectively (all P < 0.001). For identifying sarcopenia alone compared with possible sarcopenia plus non-sarcopenia, the A/L ratio achieved an AUC of 0.617 (95% CI: 0.536–0.698, P = 0.004), adiponectin reached an AUC of 0.622 (95% CI: 0.540–0.705, P = 0.002), and leptin obtained an AUC of 0.584 (95% CI: 0.501–0.668, P = 0.035). Subgroup analyses revealed that the discriminative ability of adipokines was generally better in earlier CKM stages (stages 1–2) compared with later stages (stages 3–4). Specifically, for identifying possible sarcopenia, the A/L ratio achieved an AUC of 0.643 (P < 0.001) in CKM stages 1–2, compared with an AUC of 0.589 (P = 0.024) in stages 3–4. Similarly, for identifying sarcopenia alone, the A/L ratio showed better discriminative ability in CKM stages 1–2 (AUC = 0.622, P = 0.025) than in stages 3–4 (AUC = 0.577, P = 0.204).

**Table 5:** ROC curve analysis of adipokines for identifying sarcopenia severity across CKM stages.

Variable	Group	Sensitivity (95% CI)	Specificity (95% CI)	AUC (95% CI)	P-value	Cutoff
**Ln-Adiponectin**	**Possible sarcopenia + sarcopenia vs. non-sarcopenia**					
	Total sample	0.719 (0.623−0.770)	0.530 (0.423−0.593)	0.651 (0.609−0.694)	<0.001	7.170
	CKM stages 1−2	0.534 (0.384−0.644)	0.709 (0.546−0.814)	0.632 (0.559−0.705)	0.001	10.443
	CKM stages 3−4	0.707 (0.581−0.778)	0.562 (0.425−0.639)	0.645 (0.590−0.700)	<0.001	7.153
	**Sarcopenia vs. possible sarcopenia + non-sarcopenia**					
	Total sample	0.649 (0.491−0.772)	0.595 (0.397−0.678)	0.622 (0.540−0.705)	0.002	9.822
	CKM stages 1−2	0.727 (0.515−0.849)	0.573 (0.221−0.719)	0.673 (0.567−0.780)	0.001	10.224
	CKM stages 3−4	0.542 (0.292−0.708)	0.628 (0.207−0.739)	0.525 (0.398−0.653)	0.676	9.822
**Ln-Leptin**	**Possible sarcopenia + sarcopenia vs. non-sarcopenia**					
	Total sample	0.639 (0.546−0.693)	0.574 (0.448−0.643)	0.604 (0.560−0.648)	<0.001	2.132
	CKM stages 1−2	0.569 (0.449−0.647)	0.674 (0.506−0.747)	0.605 (0.547−0.662)	<0.001	1.817
	CKM stages 3−4	0.548 (0.370−0.644)	0.581 (0.395−0.698)	0.549 (0.473−0.626)	0.210	1.183
	**Sarcopenia vs. possible sarcopenia + non-sarcopenia**					
	Total sample	0.561 (0.404−0.684)	0.628 (0.376−0.748)	0.584 (0.501−0.668)	0.035	1.100
	CKM stages 1−2	0.292 (0.083−0.458)	0.910 (0.675−0.947)	0.583 (0.456−0.710)	0.173	0.152
	CKM stages 3−4	0.485 (0.273−0.636)	0.714 (0.362−0.844)	0.556 (0.436−0.676)	0.303	0.587
**Ln-A/L ratio**	**Possible sarcopenia + sarcopenia vs. non-sarcopenia**					
	Total sample	0.724 (0.611−0.785)	0.544 (0.462−0.601)	0.641 (0.598−0.685)	<0.001	3.090
	CKM stages 1−2	0.669 (0.536−0.741)	0.612 (0.483−0.681)	0.643 (0.588−0.698)	<0.001	3.090
	CKM stages 3−4	0.862 (0.703−0.931)	0.302 (0.104−0.407)	0.589 (0.512−0.665)	0.024	2.468
	**Sarcopenia vs. possible sarcopenia + non-sarcopenia**					
	Total sample	0.404 (0.246−0.509)	0.830 (0.575−0.899)	0.617 (0.536−0.698)	0.004	29.424
	CKM stages 1−2	0.455 (0.212−0.606)	0.833 (0.475−0.899)	0.622 (0.508−0.736)	0.025	29.399
	CKM stages 3−4	0.292 (0.083−0.458)	0.901 (0.548−0.955)	0.577 (0.450−0.704)	0.204	64.269

Note: A/L ratio, adiponectin-to-leptin ratio; AUC, area under the curve; CI, confidence interval; CKM, cardiovascular-kidney-metabolic syndrome; ROC, receiver operating characteristic.

### 3.6 Sensitivity analyses

After excluding these 48 participants with isolated low muscle mass in the non-sarcopenia group, higher adiponectin (OR: 2.01, 95% CI: 1.41–2.87, *P* < 0.001) and higher A/L ratio (OR: 1.19, 95% CI: 1.03–1.37, *P* = 0.021) remained associated with increased odds of possible sarcopenia, while higher leptin remained associated with lower odds of possible sarcopenia (OR: 0.73, 95% CI: 0.53–0.99, *P* = 0.044). All associations kept consistent direction and statistical significance relative to primary analyses, confirming isolated low muscle mass in the reference group did not materially alter the main conclusions (Supplementary Table S2). To evaluate the impact of BMI adjustment on the observed associations, we re-conducted all fully adjusted models after excluding BMI as a covariate (Supplementary Tables S3–S5). The associations of all three adipokines with possible sarcopenia and low physical function remained fully consistent in both direction and statistical significance between BMI-adjusted and unadjusted models, confirming the robustness of our findings regarding adipokines and early sarcopenia-related functional decline. Notable differences were observed for skeletal muscle mass index (SMI) and confirmed sarcopenia. Leptin shifted from a significant negative association with SMI (β = −0.237, *P* < 0.001) in BMI-adjusted models to a significant positive association (β = 0.090, *P* = 0.031) without BMI adjustment, while the A/L ratio reversed from a significant positive association (β = 0.084, *P* < 0.001) to a significant negative association (β = −0.048, *P* = 0.016). Furthermore, none of the adipokines were significantly associated with confirmed sarcopenia in BMI-adjusted models, whereas all became statistically significant in unadjusted models.

To further verify result robustness across disease severity, we performed stratified analyses by CKM stage (stage 1–2 versus 3–4) and tested multiplicative interaction terms between adipokine levels and CKM status **(**Supplementary Table S6-S8). Significant associations between adipokines and sarcopenia related phenotypes were predominantly preserved among participants with early CKM (stage 1–2). Higher adiponectin and A/L ratio were linked to elevated risks of poor physical performance and probable sarcopenia, whereas elevated leptin correlated with better muscle related indicators and lower sarcopenia risk. In contrast, most associations were attenuated and lost statistical significance in advanced CKM (stage 3–4). Most P values for interaction between adipokines and CKM group were non-significant (P-interaction > 0.05).

## 4 Discussion

This cross-sectional study examined the associations of adiponectin, leptin, and the adiponectin-to-leptin ratio with sarcopenia in older adults with CKM syndrome. Adiponectin mainly correlates with impaired physical function, leptin correlates positively with reduced muscle mass and inversely with physical function, and the A/L ratio relates to all three sarcopenia related indicators. All three markers show significant associations with possible sarcopenia instead of confirmed sarcopenia after full adjustment.

The finding that higher adiponectin levels were independently associated with higher odds of low physical function is consistent with the emerging concept of the adiponectin paradox observed in aging and metabolic disease. Although adiponectin is classically regarded as a cardioprotective and insulin-sensitizing hormone [5,11], elevated circulating adiponectin in older adults frequently reflects a pathological context rather than a protective one. Meta-analytic evidence has confirmed that sarcopenia patients exhibit significantly higher adiponectin concentrations compared with non-sarcopenic individuals [15], and studies in metabolic syndrome and obesity have demonstrated a negative correlation between adiponectin and skeletal muscle mass index, even after adjustment for BMI [24]. Consistent findings have been reported in community-dwelling older women, where higher adiponectin was independently associated with lower appendicular lean mass [18], and in Japanese older adults, where adiponectin correlated inversely with skeletal muscle mass and positively with myosteatosis [17]. The predominant association of adiponectin with physical performance rather than muscle mass in the fully adjusted model suggests that adiponectin dysregulation is particularly associated with neuromuscular function and mitochondrial-dependent aerobic capacity, rather than directly related to protein synthesis and muscle mass accrual [11,25]. This interpretation is further supported by the significant associations with 5TSTS and gait speed in the linear regression analyses, both of which depend heavily on lower-limb neuromuscular power and aerobic efficiency.

Paradoxically, leptin was positively associated with low muscle mass but inversely related to impaired physical function. This association was independent of overall adiposity after adjustment for BMI and lifestyle factors. Leptin resistance, characterized by impaired leptin receptor signaling in peripheral tissues under conditions of chronic hyperleptinemia, has been proposed as a key explanation for the relationship between adipose tissue dysfunction and muscle catabolism [11]. Under leptin resistance, leptin loses its anabolic properties, and sustained pro-inflammatory activity may contribute to muscle protein degradation [5]. These findings are consistent with previous evidence from population studies showing that serum leptin is negatively associated with skeletal muscle mass index [19,20] and that hyperleptinemia contributes to sarcopenic obesity [19]. Prior imaging-based studies have also demonstrated that higher leptin is associated with lower abdominal muscle area and greater muscle radiodensity, markers of muscle quality, independent of total adiposity [21]. In contrast, the protective association of leptin with physical function warrants careful interpretation. Leptin receptors are expressed in the cerebellum, motor cortex, and basal ganglia [11], and leptin has been shown to modulate proprioception, motor coordination, and executive function relevant to physical performance in older adults. It is possible that in the context of CKM syndrome, leptin retains partial signaling activity in neuromuscular regulatory circuits even in the setting of metabolic resistance in skeletal muscle, thus preserving certain dimensions of physical performance such as gait speed and balance. An alternative explanation is that the apparent protective association partly reflects residual confounding. Individuals with very low leptin in this CKM sample may disproportionately represent those with advanced sarcopenia, severe CKD, or cardiac cachexia, all of which are associated with both low leptin and severely impaired physical performance. This possibility is consistent with prior evidence from hemodialysis cohorts, where higher leptin levels were paradoxically associated with improved survival [26], suggesting a complex, context-dependent role of leptin in advanced CKM states.

The A/L ratio demonstrated the most comprehensive and consistent associations across all sarcopenia components in the fully adjusted model, including lower odds of low muscle mass, higher odds of low muscle strength and low physical function. This breadth of association reflects the integrative nature of the A/L ratio as a biomarker of adipose tissue dysfunction, capturing the reciprocal dysregulation of both adipokines simultaneously [[12], [13], [14]]. A higher A/L ratio in this CKM cohort may signal advanced adiponectin resistance superimposed on relative leptin deficiency in the context of low body weight or accelerated adipose tissue wasting in older adults [16]. From a clinical perspective, the A/L ratio has been proposed as a superior biomarker of adipose tissue inflammation and insulin resistance compared with either adipokine alone [[12], [13], [14]], and our findings extend this utility to the domain of sarcopenic muscle function in CKM patients. Prior studies in Chinese older adults have similarly demonstrated that the leptin-to-adiponectin ratio outperforms either marker alone in identifying metabolic syndrome [27], lending additional support for the discriminative value of ratio-based adipokine metrics in this population. Extending beyond older adult cohorts, the A/L ratio has also been identified as a superior marker of insulin resistance and cardiometabolic risk compared with either adipokine alone in obese children and adolescents [28], suggesting that this ratio retains discriminative value across diverse age groups and metabolic contexts.

Notably, the associations of leptin and the A/L ratio with low muscle mass emerged only after adjustment for BMI and lifestyle factors (Model 2), whereas these associations were absent in the age- and sex-adjusted model (Model 1). This pattern suggests that the relationship between adipokines and muscle mass is partially confounded by adiposity, and that controlling for BMI unmasks the independent effect of adipokine dysregulation on sarcopenia risk. Previous studies have consistently shown that BMI is the primary determinant of circulating adiponectin and leptin levels, and that obesity itself also directly affects sarcopenia status [29,30]. We therefore included BMI as a potential confounder in our primary analyses, consistent with previous studies on adipokines and sarcopenia [19,31]. Leptin is strongly correlated with body fat mass, and elevated circulating leptin in obesity reflects leptin resistance rather than functional leptin signaling. When BMI is statistically controlled, the remaining variance in leptin levels may better capture adipose tissue dysfunction and its downstream effects on muscle metabolism. Similarly, the A/L ratio, which integrates both adiponectin and leptin into a single metric of adipose tissue function, demonstrated significant associations with all sarcopenia components only after BMI adjustment, underscoring its value as a biomarker independent of obesity status. In contrast, adiponectin showed no significant association with low muscle mass across all models, suggesting that its relationship with muscle mass, if present, may be largely mediated through or confounded by body composition. These findings align with previous studies indicating that associations between adipokines and skeletal muscle may differ between obese and non-obese individuals, and highlight the importance of considering obesity status when interpreting adipokine-sarcopenia associations. We further systematically evaluated the potential for overadjustment and collider stratification bias, two common pitfalls in covariate adjustment. Interestingly, our sensitivity analyses further clarified this heterogeneity by comparing fully adjusted models with and without BMI. As shown in Supplementary Table S3–S5, associations involving muscle mass and confirmed sarcopenia changed markedly following BMI exclusion, whereas correlations with early physical dysfunction remained stable across both model schemes. These results highlight that the relationship between adipokines and sarcopenia is highly dependent on body composition, and future studies should incorporate more precise measures of adiposity and muscle quality rather than relying solely on BMI, to better disentangle the independent associations of adipokine dysregulation across the full spectrum of sarcopenia progression.

The observation that adipokine associations with possible sarcopenia, but not with confirmed sarcopenia, remained significant after full adjustment merits discussion. Consistent with the 2019 AWGS diagnostic framework that prioritizes physical performance and muscle strength for sarcopenia screening, individuals with isolated low muscle mass alone were categorized into the non-sarcopenia group in primary analysis. Corresponding sensitivity analysis after excluding these subjects generated largely unchanged statistical outcomes. Several non-mutually exclusive explanations are plausible. First, the sample of confirmed sarcopenia participants was relatively small, limiting statistical power to detect significant associations after adjustment for a large number of covariates. Second, confirmed sarcopenia represents a later, multi-factorial phenotype in which mechanical, nutritional, inflammatory, and hormonal pathways collectively converge. In this setting, the marginal contribution of any single adipokine pathway may be attenuated by competing pathological drivers [7]. Third, given that confirmed sarcopenia is more prevalent in advanced CKM stages, the increased metabolic burden, cardiovascular complexity, and polypharmacy characteristic of these stages may mask the independent adipokine signal [2,32]. This interpretation is supported by the subgroup ROC analyses, which showed consistently lower discriminative performance for all three adipokines in CKM stages 3–4 compared with stages 1–2. In addition, among the 256 participants classified as possible sarcopenia, only 5.86% had isolated low muscle strength, 72.27% exhibited isolated impaired physical performance, and another 21.87% had concurrent deficits in both strength and physical function. Such uneven constituent composition indicates that the observed associations of adiponectin and the A/L ratio with possible sarcopenia are predominantly driven by physical function abnormalities rather than comprehensive multidimensional sarcopenia changes. Hence these relevant results should be interpreted mainly from the perspective of physical function decline instead of overall sarcopenia phenotype. Our separate analyses across individual sarcopenia components also revealed divergent associations of the adipokines with low muscle strength and low physical function (Table 3).

The superior discriminative value of adipocytokines for sarcopenia in CKM stages 1–2 compared with stages 3 to 4 has potentially clinical implications. Stratified analyses consistently confirm this stage-dependent association pattern (Supplementary Tables S6-S8). Significant associations between adipokines and all sarcopenia components as well as sarcopenia severity are largely restricted to CKM stages 1–2. Most associations are attenuated and lose statistical significance in stages 3–4, although interaction tests show no significant effect modification between adipokines and CKM stage. The lack of statistically significant interaction terms likely reflects limited statistical power due to the relatively small sample size in the advanced CKM subgroup, rather than a true absence of effect modification. In early CKM stages, adipokine dysregulation, particularly the upward shift of adiponectin and the A/L ratio, may represent an early warning signal of impending sarcopenic deterioration, which may occur before the clinical manifestation of confirmed sarcopenia or overt cardiovascular events [16,33]. As CKM syndrome advances to stages 3–4, where organ-level dysfunction dominates and adipokine-mediated mechanisms are partially supplanted by uremic toxicity, heightened inflammatory burden, and cardiac cachexia, the utility of adipokines as standalone screening tools diminishes [26,32].

Several limitations must be acknowledged. First, the cross-sectional design precludes causal inference, and longitudinal studies are needed to establish directionality. Second, the predominant representation of Han Chinese residents of Beijing limits generalizability to other ethnic or geographic populations. Third, muscle mass was assessed by bioelectrical impedance analysis rather than the reference standard of DXA or CT, which may introduce measurement variability, particularly in individuals with altered hydration states common in CKM populations. Advanced CKM patients frequently develop fluid retention and edema, which leads to systematic bias in BIA-derived skeletal muscle index measurements. Fourth, only 57 participants (9.0%) were diagnosed with confirmed sarcopenia in the present study. Such a small sample size may lead to insufficient statistical power and increase the risk of type II error. Finally, single time-point measurement of adipokines may not adequately capture the dynamic fluctuations characteristic of CKM-related metabolic states.

## 5 Conclusion

In conclusion, adiponectin, leptin, and the A/L ratio demonstrate component-specific associations with sarcopenia in older adults with CKM syndrome. These adipokines show slight-to-modest discriminative performance for sarcopenia, particularly in early CKM stages. Future prospective and mechanistic studies are warranted to clarify the causal pathways linking adipokine dysregulation to sarcopenia progression across the CKM syndrome.

## CRediT authorship contribution statement

Y Zhou contributed to the concept and design of the study and wrote the manuscript; Y Zhang and M Yang collected and interpreted the data; Z Huo and H Shi contributed to critical revision of the article; J Shen participated in the study design, contributed to methodology and supervision, and provided funding support; C Zhang contributed to methodology, supervision, and critical revision of the article. All authors have read and agreed to the published version of the article.

## Consent for publication

Not applicable.

## Ethics approval and consent to participate

The study protocol was approved by the Ethics Committee of Beijing Hospital (2022BJYYEC-260-03), and written informed consent was obtained from all participants.

## Declaration of Generative AI and AI-assisted technologies in the writing process

The authors only used AI to correct the grammar in some sentences.

## Funding

This study is supported by the National High Level Hospital Clinical Research Funding (grant numbers: BJ-2024-178; BJ-2025-240; BJ-2025-253; BJ-2022-149).

## Declaration of Competing Interest

The authors declare that they have no competing interests.

## Availability of data and materials

The datasets analyzed during the current study are available from the corresponding author on reasonable request.
