# Supplementary material for: Associations of adiponectin, leptin, and the adiponectin-to-leptin ratio with sarcopenia in older adults with cardiovascular-kidney-metabolic syndrome
Source: J Nutr Health Aging. 2026 Jul 1;30(9):100921. doi: 10.1016/j.jnha.2026.100921 (PMC13351126; doi:10.1016/j.jnha.2026.100921)
Supplement: Supplementary file 1 [file mmc1.docx]

**Supplementary materials**

**Supplementary table 1.** Comparison of demographic characteristics and sarcopenia-related indicators between enrolled participants and excluded participants without blood specimens

| **Variables** | **Included sample (N=632)** | **Excluded sample (n=233)** | **P-value** |  |
| --- | --- | --- | --- | --- |
|  |  |  |  |  |
| **Sample characteristics** |  |  |  |  |
| Age, years, mean ± SD | 70.60 ± 6.09 | 69.54 ± 6.54 | 0.036 |  |
| Female, n (%) | 359 (56.8) | 146 (62.7) | 0.119 |  |
| Han ethnicity, n (%) | 592 (93.7) | 224 (96.1) | 0.148 |  |
| Education level, n (%) |  |  | 0.676 |  |
| Primary or below | 23 (3.6) | 11 (4.7) |  |  |
| Secondary | 422 (66.8) | 158 (67.8) |  |  |
| Tertiary or above | 187 (29.6) | 64 (27.5) |  |  |
| Married and cohabiting, n (%) | 535 (84.7) | 192 (82.4) | 0.426 |  |
| BMI, kg/m², mean ± SD | 24.89 ± 3.35 | 25.28 ± 3.19 | 0.144 |  |
| Current smoking, n (%) | 73 (11.6) | 26 (11.2) | 0.872 |  |
| Current drinking, n (%) | 73 (11.6) | 21 (9.0) | 0.279 |  |
| Physical activity habit, n (%) | 540 (85.4) | 184 (78.9) | 0.025 |  |
| **Health status** |  |  |  |  |
| ADL impairment, n (%) | 39 (6.2) | 16 (6.9) | 0.679 |  |
| Cognitive impairment, n (%) | 158 (25.0) | 53 (25.4) | 0.917 |  |
| Depression, n (%) | 87 (13.8) | 49 (21.3) | 0.009 |  |
| Medication use, M (P_25_, P_75_) | 3 (2, 5) | 3 (2, 5) | 0.544 |  |
| **Sarcopenia components** |  |  |  |  |
| SMI, kg/m², mean ± SD | 6.95 ± 1.06 | 6.98 ± 1.02 | 0.681 |  |
| Handgrip strength, kg, mean ± SD | 28.25 ± 9.24 | 27.05 ± 9.85 | 0.113 |  |
| 5TSTS, s, mean ± SD | 10.99 ± 3.48 | 11.11 ± 3.67 | 0.658 |  |
| 6-m gait speed, m/s, mean ± SD | 1.03 ± 0.23 | 0.99 ± 0.25 | 0.052 |  |
| SPPB score, M (P_25_, P_75_) | 10 (8, 10) | 10 (8, 10) | 0.228 |  |

ADL, activities of daily living; A/L ratio, adiponectin-to-leptin ratio; BMI, body mass index; SMI, skeletal muscle mass index; SPPB, Short Physical Performance Battery; 5TSTS, 5-time sit-to-stand test.

**Supplementary table 2.** Multinomial logistic regression analysis of adipokines and sarcopenia severity after excluding 48 participants with isolated low muscle mass

| **Independent**  **variable** | **Possible sarcopenia vs. non-sarcopenia** | | | **Sarcopenia vs. non-sarcopenia** | | |
| --- | --- | --- | --- | --- | --- | --- |
|  | β (95% CI) | OR (95% CI) | P-value | β (95% CI) | OR (95% CI) | P-value |
| **Ln-Adiponectin** | 0.698 (0.342, 1.054) | 2.01 (1.41, 2.87) | <0.001 | 0.495 (-0.104, 1.094) | 1.64 (0.90, 2.99) | 0.105 |
| **Ln-Leptin** | -0.321 (-0.634, -0.009) | 0.73 (0.53, 0.99) | 0.044 | 0.036 (-0.545, 0.616) | 1.04 (0.58, 1.85) | 0.904 |
| **Ln-A/L ratio** | 0.170 (0.026, 0.314) | 1.19 (1.03, 1.37) | 0.021 | 0.093 (-0.157, 0.343) | 1.10 (0.85, 1.41) | 0.466 |

Adjusted for age, sex, ethnicity, education level, marital status, smoking, alcohol consumption, physical activity, BMI, ADL impairment, cognitive impairment, depressive symptoms, polypharmacy, TC, TG, HDL-C, LDL-C, FPG, and eGFR.

**Supplementary table 3.** Associations of adiponectin, leptin, and A/L ratio with sarcopenia indicators in fully adjusted models without BMI as a covariate

| **Independent variable** | **Dependent variable** | **β (95% CI)** | **P-value** |
| --- | --- | --- | --- |
| **Ln-Adiponectin** | SMI | -0.036 (-0.132, 0.060) | 0.462 |
|  | Grip strength | -0.646 (-1.447, 0.155) | 0.114 |
|  | 5TST | 0.614 (0.202, 1.026) | 0.004 |
|  | Gait speed | -0.022 (-0.048, 0.004) | 0.097 |
|  | SPPB | -0.256 (-0.434, -0.078) | 0.005 |
| **Ln-Leptin** | SMI | 0.090 (0.008, 0.172) | 0.031 |
|  | Grip strength | 0.108 (-0.578, 0.794) | 0.757 |
|  | 5TST | -0.072 (-0.427, 0.282) | 0.689 |
|  | Gait speed | 0.037 (0.015, 0.059) | 0.001 |
|  | SPPB | 0.067 (-0.086, 0.220) | 0.389 |
| **Ln-A/L ratio** | SMI | -0.048 (-0.087, -0.009) | 0.016 |
|  | Grip strength | -0.233 (-0.560, 0.093) | 0.161 |
|  | 5TST | 0.062 (-0.107, 0.230) | 0.472 |
|  | Gait speed | -0.014 (-0.024, -0.003) | 0.011 |
|  | SPPB | -0.036 (-0.107, 0.035) | 0.321 |

Adjusted for age, sex, ethnicity, education level, marital status, smoking, alcohol consumption, physical activity, ADL impairment, cognitive impairment, depressive symptoms, polypharmacy, TC, TG, HDL-C, LDL-C, FPG, and eGFR.

**Supplementary table 4.** Binary logistic regression analysis of adipokines and sarcopenia components in fully adjusted models without BMI as a covariate

| **Independent variable** | **Dependent variable** | **β (95% CI)** | **OR (95% CI)** | **P-value** |
| --- | --- | --- | --- | --- |
| **Ln-Adiponectin** | Low muscle mass | 0.083 (-0.287, 0.461) | 1.09 (0.75, 1.59) | 0.664 |
|  | Low muscle strength | 0.433 (0.016, 0.866) | 1.54 (1.02, 2.38) | 0.045 |
|  | Low physical function | 0.718 (0.392, 1.056) | 2.05 (1.48, 2.87) | <0.001 |
| **Ln-Leptin** | Low muscle mass | -0.237 (-0.574, 0.089) | 0.79 (0.56, 1.09) | 0.160 |
|  | Low muscle strength | -0.325 (-0.686, 0.024) | 0.72 (0.50, 1.02) | 0.072 |
|  | Low physical function | -0.203 (-0.468, 0.060) | 0.82 (0.63, 1.06) | 0.131 |
| **Ln-A/L ratio** | Low muscle mass | 0.132 (-0.026, 0.286) | 1.14 (0.97, 1.33) | 0.096 |
|  | Low muscle strength | 0.222 (0.061, 0.381) | 1.25 (1.06, 1.46) | 0.006 |
|  | Low physical function | 0.131 (0.008, 0.255) | 1.14 (1.01, 1.29) | 0.038 |

Adjusted for age, sex, ethnicity, education level, marital status, smoking, alcohol consumption, physical activity, ADL impairment, cognitive impairment, depressive symptoms, polypharmacy, TC, TG, HDL-C, LDL-C, FPG, and eGFR.

**Supplementary table 5.** Multinomial logistic regression analysis of adipokines and sarcopenia severity in fully adjusted models without BMI as a covariate

| **Independent**  **variable** | **Possible sarcopenia vs. non-sarcopenia** | | | **Sarcopenia vs. non-sarcopenia** | | |
| --- | --- | --- | --- | --- | --- | --- |
|  | β (95% CI) | OR (95% CI) | P-value | β (95% CI) | OR (95% CI) | P-value |
| Ln-Adiponectin | 0.734 (0.384, 1.085) | 2.08 (1.47, 2.96) | <0.001 | 0.669 (0.110, 1.228) | 1.95 (1.12, 3.41) | 0.019 |
| Ln-Leptin | -0.450 (-0.756, -0.144) | 0.64 (0.47, 0.87) | 0.004 | -0.639 (-1.132, -0.145) | 0.53 (0.32, 0.86) | 0.011 |
| Ln-A/L ratio | 0.223 (0.082, 0.365) | 1.25 (1.09, 1.44) | 0.002 | 0.355 (0.147, 0.564) | 1.43 (1.16, 1.76) | <0.001 |

Adjusted for age, sex, ethnicity, education level, marital status, smoking, alcohol consumption, physical activity, ADL impairment, cognitive impairment, depressive symptoms, polypharmacy, TC, TG, HDL-C, LDL-C, FPG, and eGFR.

**Supplementary table 6.** Associations of adiponectin, leptin, and A/L ratio with sarcopenia indicators across different CKM stages

| **Independent**  **variable** | **Dependent**  **variable** | **CKM stages 1-2** | |  | **CKM stages 3-4** | | **P-interaction** |
| --- | --- | --- | --- | --- | --- | --- | --- |
|  |  | **β (95% CI)** | **P-value** |  | **β (95% CI)** | **P-value** |  |
| **Ln-Adiponectin** | SMI | 0.054 (-0.024, 0.133) | 0.174 |  | -0.027 (-0.159, 0.105) | 0.690 | 0.512 |
|  | Grip strength | -0.239 (-1.237, 0.758) | 0.637 |  | -1.113 (-2.554, 0.329) | 0.130 | 0.611 |
|  | 5TST | 0.690 (0.234, 1.146) | 0.003 |  | 0.390 (-0.453, 1.232) | 0.363 | 0.831 |
|  | Gait speed | -0.033 (-0.064, -0.001) | 0.041 |  | -0.003 (-0.050, 0.044) | 0.910 | 0.060 |
|  | SPPB | -0.285 (-0.479, -0.090) | 0.004 |  | -0.172 (-0.554, 0.210) | 0.376 | 0.915 |
| **Ln-Leptin** | SMI | -0.229 (-0.301, -0.156) | <0.001 |  | -0.236 (-0.343, -0.129) | <0.001 | 0.606 |
|  | Grip strength | -0.136 (-1.102, 0.830) | 0.782 |  | -0.683 (-1.903, 0.537) | 0.271 | 0.495 |
|  | 5TST | -0.669 (-1.109, -0.229) | 0.003 |  | -0.249 (-0.959, 0.461) | 0.491 | 0.288 |
|  | Gait speed | 0.074 (0.044, 0.103) | <0.001 |  | 0.028 (-0.011, 0.068) | 0.158 | 0.147 |
|  | SPPB | 0.290 (0.103, 0.478) | 0.003 |  | 0.005 (-0.317, 0.328) | 0.974 | 0.355 |
| **Ln-A/L ratio** | SMI | 0.099 (0.065, 0.133) | <0.001 |  | 0.064 (0.013, 0.115) | 0.014 | 0.083 |
|  | Grip strength | 0.001 (-0.449, 0.450) | 0.895 |  | -0.072 (-0.648, 0.505) | 0.807 | 0.918 |
|  | 5TST | 0.231 (0.025, 0.437) | 0.028 |  | 0.213 (-0.120, 0.545) | 0.210 | 0.727 |
|  | Gait speed | -0.022 (-0.036, -0.008) | 0.002 |  | -0.017 (-0.036, 0.001) | 0.071 | 0.572 |
|  | SPPB | -0.105 (-0.189, -0.020) | 0.015 |  | -0.011 (-0.162, 0.140) | 0.885 | 0.520 |

Adjusted for age, sex, ethnicity, education level, marital status, smoking, alcohol consumption, physical activity, BMI, ADL impairment, cognitive impairment, depressive symptoms, polypharmacy, TC, TG, HDL-C, LDL-C, FPG, and eGFR.

**Supplementary table 7.** Binary logistic regression analysis of adipokines and sarcopenia components across different CKM stages

| **Independent**  **variable** | **Dependent**  **variable** | **CKM stages 1-2** | | |  | **CKM stages 3-4** | | | **P-interaction** |
| --- | --- | --- | --- | --- | --- | --- | --- | --- | --- |
|  |  | **β (95% CI)** | **OR (95% CI)** | **P-value** |  | **β (95% CI)** | **OR (95% CI)** | **P-value** |  |
| **Ln-Adiponectin** | Low muscle mass | -0.479 (-1.058, 0.087) | 0.62 (0.35, 1.09) | 0.099 |  | 0.692 (-0.264, 1.721) | 2.00 (0.77, 5.59) | 0.168 | 0.064 |
|  | Low muscle strength | 0.192 (-0.328, 0.730) | 1.21 (0.72, 2.07) | 0.473 |  | 0.908 (0.070, 1.801) | 2.48 (1.07, 6.05) | 0.039 | 0.060 |
|  | Low physical function | 0.895 (0.475, 1.338) | 2.45 (1.61, 3.81) | <0.001 |  | 0.433 (-0.211, 1.100) | 1.54 (0.81, 3.00) | 0.193 | 0.652 |
| **Ln-Leptin** | Low muscle mass | 1.042 (0.423, 1.707) | 2.83 (1.53, 5.51) | 0.001 |  | 0.215 (-0.624, 1.044) | 1.24 (0.54, 2.84) | 0.611 | 0.425 |
|  | Low muscle strength | -0.534 (-1.074, -0.020) | 0.59 (0.34, 0.98) | 0.046 |  | -0.239 (-0.935, 0.429) | 0.79 (0.39, 1.54) | 0.489 | 0.719 |
|  | Low physical function | -0.602 (-0.995, -0.220) | 0.55 (0.37, 0.80) | 0.002 |  | -0.114 (-0.674, 0.445) | 0.89 (0.51, 1.56) | 0.688 | 0.948 |
| **Ln-A/L ratio** | Low muscle mass | -0.414 (-0.717, -0.141) | 0.66 (0.49, 0.87) | 0.005 |  | 0.107 (-0.269, 0.461) | 1.11 (0.76, 1.58) | 0.561 | 0.112 |
|  | Low muscle strength | 0.160 (-0.069, 0.386) | 1.17 (0.93, 1.47) | 0.165 |  | 0.378 (0.068, 0.694) | 1.46 (1.07, 2.00) | 0.017 | 0.215 |
|  | Low physical function | 0.274 (0.101, 0.454) | 1.31 (1.11, 1.57) | 0.002 |  | 0.115 (-0.129, 0.366) | 1.12 (0.88, 1.44) | 0.359 | 0.991 |

Adjusted for age, sex, ethnicity, education level, marital status, smoking, alcohol consumption, physical activity, BMI, ADL impairment, cognitive impairment, depressive symptoms, polypharmacy, TC, TG, HDL-C, LDL-C, FPG, and eGFR.

**Supplementary table 8.** Multinomial logistic regression analysis of adipokines and sarcopenia severity across different CKM stages

| **Independent variable** | | **CKM stages 1-2** | | |  | **CKM stages 3-4** | | | **P-interaction** |
| --- | --- | --- | --- | --- | --- | --- | --- | --- | --- |
|  |  | **β (95% CI)** | **OR (95% CI)** | **P-value** |  | **β (95% CI)** | **OR (95% CI)** | **P-value** |  |
| **Ln-Adiponectin** |  |  |  |  |  |  |  |  | 0.074 |
| Possible sarcopenia + sarcopenia vs. non-sarcopenia | | 0.972 (0.523, 1.422) | 2.64 (1.69, 4.15) | <0.001 |  | 0.299 (-0.370, 0.968) | 1.35 (0.69, 2.63) | 0.380 |  |
| Sarcopenia vs. possible sarcopenia + non-sarcopenia | | 0.346 (-0.437, 1.129) | 1.41 (0.65, 3.09) | 0.387 |  | 0.735 (-0.433, 1.904) | 2.09 (0.65, 6.71) | 0.217 |  |
| **Ln-Leptin** |  |  |  |  |  |  |  |  | 0.898 |
| Possible sarcopenia + sarcopenia vs. non-sarcopenia | | -0.684 (-1.094, -0.274) | 0.50 (0.33, 0.76) | 0.001 |  | -0.071 (-0.641, 0.498) | 0.93 (0.53, 1.65) | 0.806 |  |
| Sarcopenia vs. possible sarcopenia + non-sarcopenia | | -0.070 (-0.833, 0.694) | 0.93 (0.43, 2.00) | 0.858 |  | -0.263 (-1.240, 0.713) | 0.77 (0.29, 2.04) | 0.597 |  |
| **Ln-A/L ratio** |  |  |  |  |  |  |  |  | 0.690 |
| Possible sarcopenia + sarcopenia vs. non-sarcopenia | | 0.297 (0.109, 0.485) | 1.35 (1.12, 1.62) | 0.002 |  | 0.126 (-0.131, 0.383) | 1.13 (0.88, 1.47) | 0.338 |  |
| Sarcopenia vs. possible sarcopenia + non-sarcopenia | | 0.081 (-0.237, 0.398) | 1.08 (0.79, 1.49) | 0.619 |  | 0.338 (-0.088, 0.764) | 1.40 (0.92, 2.15) | 0.119 |  |

Adjusted for age, sex, ethnicity, education level, marital status, smoking, alcohol consumption, physical activity, BMI, ADL impairment, cognitive impairment, depressive symptoms, polypharmacy, TC, TG, HDL-C, LDL-C, FPG, and eGFR.
